# Genome Sequence of an H9N2 Avian Influenza Virus Strain with Hemagglutinin-Neuraminidase Combination, Isolated from a Quail in Guangxi, Southern China

**DOI:** 10.1128/genomeA.00965-17

**Published:** 2017-09-21

**Authors:** Liji Xie, Zhixun Xie, Dan Li, Sisi Luo, Minxiu Zhang, Li Huang, Zhiqin Xie, Jiaoling Huang, Yanfang Zhang, Tingting Zeng, Xianwen Deng

**Affiliations:** Guangxi Key Laboratory of Veterinary Biotechnology, Guangxi Veterinary Research Institute, Nanning, Guangxi, China

## Abstract

We isolated a strain of H9N2 avian influenza virus from a quail in southern China in May 2015 and named it A/quail/Guangxi/198Q39/2015. All eight gene segments of the strain were sequenced. Sequence analysis indicated that the amino acid motif of the hemagglutinin cleavage site of this H9N2 virus was RSSR↓GLF, which is a typical characteristic of the low pathogenic avian influenza virus. This study will help in better understanding the epidemiology and molecular characteristics of avian influenza virus in wild birds.

## GENOME ANNOUNCEMENT

The avian influenza virus is a negative-sense, segmented RNA virus that belongs to the genus Influenza type A virus of the family *Orthomyxoviridae* (1, 2). At present, there are 18 hemagglutinin (HA) and 11 neuraminidase (NA) subtypes of avian influenza virus based on the antigenic differences of the HA and NA proteins, which are surface glycoproteins on the viral envelope (3, 4).

The H9N2 subtype avian influenza virus is widespread in the world and is the most prevalent subtype of avian influenza viruses reported in China over the past decade (5, 6). Although H9N2 is characterized as a low-pathogenic avian influenza virus, occasional infections of humans (7–10) have caused great concerns. So far, H9N2 subtype avian influenza viruses are isolated mainly from domestic birds (11); however, wildfowl and shorebirds are the natural hosts of avian influenza, virus and they facilitate the transmission of avian influenza (6, 12).

An H9N2 subtype avian influenza virus was isolated from a quail in Guangxi, China, in May 2015 and named A/quail/Guangxi/198Q39/2015 (H9N2). All eight gene segments were amplified by reverse transcription-PCR using avian influenza virus universal primers (13, 14). The amplified products were gel purified and cloned into the pMD-18T vector (TaKaRa, Dalian, China) and sequenced (TaKaRa). The sequences were assembled using the SeqMan program and manually edited to generate the final full-length genome sequence.

The complete genome of the A/quail/Guangxi/198Q39/2015 strain consists of eight segments of the HA, NA, NS, M, NP, PA, PB1, and PB2 genes. The full lengths of these segments are 1,742, 1,457, 890, 1,027, 1,565, 2,233, 2,341, and 2,341 nucleotides, respectively. The amino acid (aa) lengths of the proteins encoded by the eight genes are 560 aa (HA), 466 aa (NA), 121 aa (NS2), 217 aa (NS1), 252 aa (M1), 97 aa (M2), 498 aa (NP), 716 aa (PA), 758 aa (PB1), and 259 aa (PB2).

The amino acid residues at the cleavage site (aa 335 to 341) of the HA molecule are RSSR↓GLF, which is characteristic of low-pathogenic avian influenza virus. Sequence analysis revealed that the nucleotide sequences of the HA and NA genes of the A/quail/Guangxi/198Q39/2015 strain both belong to the Eurasian lineage. The A/quail/Guangxi/198Q39/2015 strain has Leu234 and Gly236 at the receptor-binding site in the HA protein, which suggests that it might have the ability to bind a sialic acid–2,6-NeuAcGal linkage and might have the potential to infect humans (9).

The nucleotide homology comparisons revealed that the HA gene of this strain shared the highest sequence homology (98%) with the HA gene of a Beijing avian influenza virus strain, A/chicken/Beijing/0309/2013 (GenBank accession number KM609599). The NA gene shared the highest sequence homology (98%) with A/turtledove/Guangxi/49B6/2013 (GenBank accession number KJ725014). These results are useful for future analyses of the molecular epidemiology and evolutionary characteristics of avian influenza virus.

### Accession number(s).

The genome sequence of A/quail/Guangxi/198Q39/2015 (H9N2) was deposited in GenBank under the accession numbers MF425642 to MF425649.

## References

[1] Webster RG, Bean WJ, Gorman OT, Chambers TM, Kawaoka Y 1992 Evolution and ecology of influenza A viruses. Microbiol Rev 56:152–179. 1579108 10.1128/mr.56.1.152-179.1992 PMC372859

[2] Xu Q, Xie Z, Xie L, Xie Z, Deng X, Liu J, Luo S 2014 Characterization of an avian influenza virus H9N2 strain isolated from a wild bird in southern China. Genome Announc 2(3):e00600-14. doi:10.1128/genomeA.00600-14. 24948768 PMC4064032

[3] Wu Y, Wu Y, Tefsen B, Shi Y, Gao GF 2014 Bat-derived influenza-like viruses H17N10 and H18N11. Trends Microbiol 22:183–191. doi:10.1016/j.tim.2014.01.010. 24582528 PMC7127364

[4] Tong S, Zhu X, Li Y, Shi M, Zhang J, Bourgeois M, Yang H, Chen X, Recuenco S, Gomez J, Chen LM, Johnson A, Tao Y, Dreyfus C, Yu W, McBride R, Carney PJ, Gilbert AT, Chang J, Guo Z, Davis CT, Paulson JC, Stevens J, Rupprecht CE, Holmes EC, Wilson IA, Donis RO 2013 New World bats harbor diverse influenza A viruses. PLoS Pathog 9:e1003657. doi:10.1371/journal.ppat.1003657. 24130481 PMC3794996

[5] Ji K, Jiang WM, Liu S, Chen JM, Chen J, Hou GY, Li JP, Huang BX 2010 Characterization of the hemagglutinin gene of subtype H9 avian influenza viruses isolated in 2007–2009 in China. J Virol Methods 163:186–189. doi:10.1016/j.jviromet.2009.09.013. 19781574

[6] Peng Y, Xie ZX, Liu JB, Pang YS, Deng XW, Xie ZQ, Xie LJ, Fan Q, Luo SS 2013 Epidemiological surveillance of low pathogenic avian influenza virus (LPAIV) from poultry in Guangxi Province, southern China. PLoS One 8:e77132. doi:10.1371/journal.pone.0077132. 24204754 PMC3813733

[7] Alexander DJ, Brown IH 2000 Recent zoonoses caused by influenza A viruses. Rev Sci Tech 19:197–225. 11189716 10.20506/rst.19.1.1220

[8] Peiris M, Yuen KY, Leung CW, Chan KH, Ip PL, Lai RW, Orr WK, Shortridge KF 1999 Human infection with influenza H9N2. Lancet 354:916–917. doi:10.1016/S0140-6736(99)03311-5. 10489954

[9] Wan H, Perez DR 2007 Amino acid 226 in the hemagglutinin of H9N2 influenza viruses determines cell tropism and replication in human airway epithelial cells. J Virol 81:5181–5191. doi:10.1128/JVI.02827-06. 17344280 PMC1900221

[10] Xie Z, Dong J, Tang X, Liu J, Khan MI, Pang Y, Deng X, Xie Z 2008 Sequence and phylogenetic analysis of three isolates of avian influenza H9N2 from chickens in southern China. Sch Res Exch 2008:802317. doi:10.3814/2008/802317.

[11] Olsen B, Munster VJ, Wallensten A, Waldenström J, Osterhaus ADME, Fouchier RAM 2006 Global patterns of influenza A virus in wild birds. Science 312:384–388. doi:10.1126/science.1122438. 16627734

[12] Xu Q, Xie Z, Xie L, Xie Z, Deng X, Liu J, Luo S 2014 Characterization of an avian influenza virus H9N2 strain isolated from a wild bird in southern China. Genome Announc 2(3):e00600-14. doi:10.1128/genomeA.00600-14. 24948768 PMC4064032

[13] Hoffmann E, Stech J, Guan Y, Webster RG, Perez DR 2001 Universal primer set for the full-length amplification of all influenza A viruses. Arch Virol 146:2275–2289. doi:10.1007/s007050170002. 11811679

[14] He CQ, Xie ZX, Han GZ, Dong JB, Wang D, Liu JB, Ma LY, Tang XF, Liu XP, Pang YS, Li GR 2009 Homologous recombination as an evolutionary force in the avian influenza A virus. Mol Biol Evol 26:177–187. doi:10.1093/molbev/msn238. 18931384

